# Morbidity and mortality in elderly dogs – a model for human aging

**DOI:** 10.1186/s12917-022-03518-8

**Published:** 2022-12-29

**Authors:** Patrícia Dias-Pereira

**Affiliations:** Institute of Biomedical Sciences Abel Salazar, University of Porto (ICBAS-UP), Porto, Portugal

**Keywords:** Animal model, Canine, Morbidity, Mortality, Aging, Geropathology, Necropsy, One health

## Abstract

Over the last decades, canines have experienced a marked increase in their lifespan, mirroring human populations. Several authors have pointed out the domestic dog as a suitable animal model for geropathology translational research. The aim of this study is to assess age-related morbidities and mortality in a population of 269 elderly canines (130 males and 139 females) submitted to necropsy. The organic systems exhibiting the higher number of age-related morbidities were the reproductive, cardiovascular and urinary systems and, in females, also the mammary gland. The prevalence of cardiovascular and urinary disease was significantly higher in males and mammary lesions were exclusively found in females. Urinary disease was more frequent in small breeds dogs, while peritoneum and male genital morbidities were significantly higher in larger breeds. Hyperplastic and degenerative lesions were common morbidities found in this elderly dog population. The main cause of death was neoplasia, which accounted for almost half of the deaths. Cardiovascular and urinary pathology also emerged as a frequent cause of mortality. These findings partially parallel data obtained for human species, displaying cancer and cardiovascular pathology as major causes of disease and death in elderlies. Our data reinforce the potential of the domestic dog for further translational investigations on gerontology, meeting the concept of One Health.

**Supplementary Information:**

The online version contains supplementary material available at 10.1186/s12917-022-03518-8.

## Introduction

Over the last 100 years humans have accomplished a marked increase in longevity, especially in developed countries [1, 2]. Similarly, the past few decades have witnessed marked changes in nutritional, hygienic, health and medical care for pets, reflecting in a significant increase in their lifespan [3]. This increased longevity is accompanied by the development of morphofunctional changes throughout life, resulting in an increase in age-related lesions and diseases in companion animals [1, 3]. In fact, besides cognitive and behavioural changes, several phenotypical alterations are recognized in aged dogs. These include modifications in coat/integument, impaired major organic functions (renal, hepatic, cardiac and lung function), as well as an increased prevalence of several conditions, namely neoplasms, dental and ocular disease, and musculoskeletal disorders [4, 5]. Despite this, aging is a complex multifactorial process involving many uncertainties, so that large scale studies on aging in dogs were launched aiming to unravel the phenotypical, functional, genetic and environmental mechanisms underneath this biological process [6, 7].

During the last decades, dogs and cats became “full members” of human families, sharing the owners’ environment and being exposed to the same environmental factors and pathological stimuli (infectious agents, inflammatory influences, carcinogenic conditions, and others) as humans. Indeed, pets experience several spontaneously occurring diseases comparable to those observed in humans [1, 2, 4, 8]. The relevance of human and animal health, together with the environment they share, was recently recognized and transposed into the concept of One Health.

In the last years several publications have pointed out companion animals as suitable translational models for geropathology research [1, 2, 4, 5, 8–10]. The characterization of the aging process is of uttermost importance for surveillance, early diagnosis, assessment and establishment of preventive strategies on age-related conditions, aiming health promotion in both human and canine elderly populations, in agreement with the One Health concept.

Some studies were previously performed regarding morbidity and mortality in different canine populations, which allowed a better understanding on their spectrum of diseases and causes of death. However, most of them are based solely on medical records or information provided by insurance companies and are not supported by systematic necropsy exams or histopathological analyses, which represents a considerable pitfall [11–16].

The aim of this study is to assess age-related morbidities and mortality in a population of elderly canines submitted to necropsy.

## Results

The cohort included in this retrospective survey consisted of 269 canines (130 males and 139 females). Fifty-two percent of the dogs were mixed breed and the remaining were distributed among 31 different breeds, the most represented being Poodles (8.6%), Boxers and Cocker Spaniels (5.6% each) and Labrador Retrievers (4.1%). Regarding body weight, 34.3% of the dogs weighed < 10 Kgs, 23.2% 10-20Kgs and 42.4% > 20Kgs (Table 1). Four cases (2 male and 2 females) were excluded from the analysis of data due to the advanced putrefaction of the cadaver, which compromised the collection of reliable information during the necropsy.

**Table 1:** Characterization of the canine population by gender, breed and body weight

	Total (%) *n* = 269	Males *n* = 130	Females *n* = 139
**Breed**			
Mongrel	142 (52.7)	69	73
Boxer	15 (5.6)	8	7
Cocker Spaniel	15 (5.6)	6	9
Dalmatian	4 (1.5)	2	2
Labrador Retriever	11 (4.1)	6	5
German Shepard	8 (3.0)	5	3
Pekingese	4 (1.5)	1	3
Pit Bull	4 (1.5)	1	3
Poodle	23 (8.6)	10	13
Siberian Husky	9 (3.3)	4	5
Spanish Breton	3 (1.1)	2	1
Others	54 (20.1)	26	28
**Weight**			
< 10 Kgs	68 (34.3)	34	34
10–20 Kgs	46 (23.2)	20	26
> 20 Kgs	84 (42.4)	46	38

## Morbidity

Table 2 summarizes the distribution of morbidities by organic system, according to the animal gender and body size.

**Table 2:** Distribution of morbidities by organic system, according to sex and body size

		Sex		Body size			
	Total (%) *n* = 265	Males *n* = 129	Females *n* = 136	*P* value	≤20 kg *n* = 114	> 20 kg *n* = 84	*P* value
Cardiovascular	117 (44.2)	68	49	**0.006**	48	42	0.270
Digestive	84 (31.7)	47	37	0.106	43	21	**0.058**
Endocrine	36 (13.6)	15	21	0.365	14	11	0.864
Genital – male^a^	56 (72.7)	56	–	0.243	24	23	**0.047**
Genital – female^a^	19 (61.3)	–	19		10	4	0.276
Hematopoietic	68 (25.7)	40	28	**0.052**	23	27	**0.055**
Liver	75 (28.3)	30	45	0.075	32	21	0.629
Mammary gland	47 (34.6)	0	47	**0.000**	20	12	0.856
Musculoskeletal	40 (15.1)	18	22	0.613	19	12	0.648
Nervous	9 (3.4)	3	6	0.348	5	3	0.773
Pancreas	18 (6.8)	8	10	0.709	11	4	0.198
Peritoneum	29 (10.9)	13	16	0.660	3	13	**0.001**
Respiratory	71 (26.8)	30	41	0.205	33	26	0.760
Skin/subcutaneous	62 (23.4)	34	28	0.267	27	23	0.554
Urinary	121 (45.7)	67	54	**0.046**	66	31	**0.003**

*Notes:* ^a^ = only non-neutered animals were considered (males *n* = 77; females *n* = 31); −-- not applicable

In this study, the organic system with the higher number of morbidities was the reproductive system. Genital lesions were observed in 72.7% of intact males (56/77) and in 61.3% of intact females (19/31). Lesions in the urinary and cardiovascular systems were also highly prevalent, being found in 45.7% and in 44.2% of the animals, respectively. Mammary lesions were registered in 34.6% of the females included in this study. Morbidities in the digestive tract and in the liver were found in 31.7% and in 28.3% of the animals. On the other hand, pancreatic and nervous lesions were the least frequent in this population, being registered in 6.8 and 3.4% of the animals, respectively.

As displayed in Table 2, the organic system that presented more lesions in males was the genital tract (with changes recorded in almost 73% of non-castrated male dogs), followed by the cardiovascular and urinary systems, with injuries observed in 52.7 and 51.9% of males, respectively. In females, the organic system with the highest number of changes was the urinary tract, followed by cardiovascular and mammary gland, with lesions registered in 39.7, 36 and 34.6% of the animals, respectively. The prevalence of pathological changes on the cardiovascular and urinary systems was significantly higher in males when compared to the female population (*p* = 0.006 and *p* = 0.046, respectively). Moreover, a higher number of lesions of the hematopoietic system was observed in males than in females, although the difference was not statistically significant (*p* = 0.052).

Both in the universe of dogs ≤20 kg and in > 20 kg, most of the lesions were observed in the urinary, cardiovascular and male reproductive systems. However, large breed dogs (> 20kgs) had a higher prevalence of pathology in the male reproductive tract and in the peritoneum (*p* = 0.048 and *p* = 0.001, respectively), while small breeds had a significantly higher number of urinary tract lesions (*p* = 0.003). Besides, digestive pathology was more frequent in small breed dogs, while lesions in the hematopoietic system were more prevalent in large dogs, although these differences were not statistically significant (*p* = 0.058 and *p* = 0.055, respectively).

Testicular neoplasms and prostatic hyperplasia were the most frequent morbidities found in the reproductive tract of intact males, accounting for 97.4 and 89.2% of the lesions observed in those structures, respectively. Polycystic ovaries represented the most common lesion found in ovaries of intact females (91.7%), while leiomyomas and cystic endometrial hyperplasia accounted, each, for 33.3% of the uterine/vaginal lesions of non-neutered females. All the mammary lesions registered in this canine population consisted of neoplastic lesions, 48.9% classified histologically as malignant and 51.1% as benign neoplasms.

The most common condition observed in the urinary tract was chronic kidney disease, (characterized by renal atrophy, irregular outline, and capsular adhesions, along with a histological picture of tubular/glomerular injury, interstitial inflammation, and fibrosis), which comprised 72.3% of the lesions observed in this system, followed by cystitis (12.4%).

Cardiac dilation (mainly affecting the left ventricular chamber) accounted for 36.6% of the cardiac lesions and valvular endocardiosis (mostly in the mitral valve) for 32.3%, while endocardial fibrosis/mineralization represented 10.4% of the morbidities registered in the cardiovascular system.

Neoplasias were the most common lesions found in the skin (86.6%), the nervous system (44.4%) and the digestive tract (31.7%), hyperplastic changes were more frequent in the pancreas (66.7%), endocrine organs (54.1%), liver (45.6%) and hematopoietic system (37.3%), while degenerative conditions predominated in the musculoskeletal system (38%).

## Mortality

In 6 cases it was not possible to determine the pathological condition related to the animal death, being thus classified as inconclusive and excluded from further data analysis.

In this canine population, malignant neoplasms were the leading cause of death (46.3%), followed by old age (18.2%). Cardiovascular failure and inflammatory processes accounted for 17.0 and 14.7% of the deaths, respectively. Malignant neoplasms represented the most common condition related to canine’s death, irrespective of their sex or body size. Nonetheless, the statistical analysis of data revealed that neoplastic-related deaths were significantly more frequent in large-breed dogs (*p* = 0.019), while those due to inflammatory processes were more common in smaller ones (*p* = 0.002) (Table 3).

**Table 3:** Mortality causes, according to sex and body size

		Sex			Body size		
	Total (%) *n* = 259	Males *n* = 127	Females *n* = 132	*P* value	≤20 kg *n* = 109	> 20 kg *n* = 81	*P* value
CV failure	44 (17.0)	25	19	0.257	20	16	0.807
Inflammation	38 (14.7)	20	18	0.631	22	4	**0.002**
Old age	47 (18.2)	27	20	0.202	22	14	0.614
Neoplasia	120 (46.3)	52	68	0.088	42	45	**0.019**
Trauma	5 (1.9)	1	4	–	1	0	–
Others	5 (1.9)	2	3	–	2	2	–

*Notes: CV* Cardiovascular; −-- not applicable (low number of counts)

As displayed in Table 4, the organic system most often involved in the animal death was the cardiovascular (21.8%), followed by the urinary system (11.4%) and the mammary gland (11.3%). On the contrary, the pancreas and the peritoneum represented the systems less often implicated in the animal’s death. In males the main systems involved in death were the cardiovascular, followed by the urinary and hematopoietic, while in females most of the death-related lesions located in the mammary gland followed by the cardiovascular system. Both in the group of dogs ≤20 kg and in > 20 kg, most of the death-related lesions were observed in the cardiovascular system. The multiplicity of categories defined for the organic system and the small number of observations included in each of them, limited the statistical analysis of the data. Even though, a statistically significant association was identified between urinary pathology and body size, demonstrating that lesions in this system were more often related to the animal death in the group of dogs under 20 kg (*p* = 0.002).

**Table 4:** Distribution of mortality by organic system, according to sex and body size

		Sex			Body size		
	Total % (*n* = 211)	Males (*n* = 99)	Females (*n* = 112)	*P* value	≤20 kg (*n* = 87)	> 20 kg (*n* = 66)	*P* value
Cardiovascular	46 (21.8)	25	21	0.249	18	17	0.459
Digestive	20 (9.5)	10	10	0.981	8	3	0.270
Endocrine	8 (3.8)	6	2	0.104	3	1	–
Genital	7 (3.3)	4	3	–	3	2	–
Hematopoietic	20 (9.5)	13	7	0.087	8	10	0.257
Liver	12 (5.7)	4	8	0.330	3	2	–
Mammary gland	24 (11.3)	0	24	**0.000**	9	9	0.531
Musculoskeletal	11 (5.2)	3	8	0.179	5	1	0.181
Nervous	7 (3.3)	3	4	–	4	2	–
Pancreas	3 (1.4)	1	2	–	1	1	–
Peritoneum	5 (2.4)	4	1	–	1	2	–
Respiratory	12 (5.7)	5	7	0.707	4	8	0.086
Skin/subcutaneous	12 (5.7)	7	5	0.414	3	6	0.141
Urinary	24 (11.4)	14	10	0.232	17	2	**0.002**

*Notes:* --- not applicable (low number of counts)

Most malignant neoplasms involved the mammary gland (20.2%), the hematopoietic (16.8%) and digestive (12.6%) systems. Skin/subcutaneous tissue and liver accounted for 9.2 and 8.4% of the malignant neoplasms in this canine population, respectively. When considering only male dogs, malignant neoplasms were found mainly in the hematopoietic (25.5%), digestive system (15.7%) and skin (13.7%), while in females they involved mostly the mammary gland (35.3%), followed by the digestive (10.3%) and the hematopoietic (10.3%) systems.

Nearly all canines whose death was related with old age were euthanized upon the owner’s request (92.3%) due to signs of neurologic and/or musculoskeletal disease. The owners refer blindness, deafness, incontinence, disorientation, decrease in social interactions, ataxia, erratic locomotion pattern, and poor quality of life, a clinical picture characteristic of old dogs and recognized by some authors under the designation of “cognitive dysfunction syndrome”, which was considered a natural model for Alzheimer’s disease in humans [3, 17, 18].

Most (57.9%) of the inflammatory processes related to death involved the urinary tract, followed by the respiratory (10.5%) and genital (7.9%) systems. The majority of cases of cardiovascular failure involved in the animal’s death were secondary to dilated cardiomyopathy (72.7%) or cavitary hemorrhages, namely hemothorax, hemopericardium or hemoperitoneum (13.6%).

## Discussion

This study provides a valuable picture of the most common morbidities and causes of death in a defined elderly canine population. It is based on data obtained from necropsy examinations, thus overcoming one of the main fragilities of previous papers on canine’s morbidity and mortality, which lay on data collected from medical or animal insurance companies’ records.

In the population included in this study, the organic systems that most frequently exhibited pathological changes were the reproductive, cardiovascular and urinary systems and, in females, also the mammary gland. These results overlap those from Fleming and collaborators [16] who described an association between increasing age and increased risk of cardiovascular and genitourinary disease in canines. The prevalence of cardiovascular and urinary disease was significantly higher in males than in females. In a previous investigation on a large group of canines up to 10 years of age, Bonnet and colleagues [13] also reported a higher frequency of cardiovascular disease in male dogs. The present results strengthen that data, demonstrating that this sexual predisposition is maintained with aging. The incidence of cardiovascular disease is also high in humans, especially in men [19, 20]. However, it is noteworthy that the specific cardiovascular diseases occurring in these species are different. Most of the cardiovascular morbidity and mortality in aging humans is related to vascular atherosclerotic and ischemic disease in the heart and brain, which is a very rare event in dogs [21]. On the other hand, age-related valvular disease (and consequent cardiac failure) is far more common in canines than in humans [22]. Whether these discrepancies represent species-related different physiological mechanisms of aging or differences in the interplay between age and other features (such as diet, medication, habits and lifestyle) remains to be clarified. Interestingly, in this study the prevalence of cardiac dilation is slightly higher than that of valvular endocardiosis. The reduced number of small dogs included, representing nearly 34% of the total population, may help to explain the lower number of cases of valvular endocardiosis (a typical condition of small breed geriatric dogs) compared to dilated cardiomyopathy (a disease more frequent in large breed animals).Urinary disease was more frequent in small breeds dogs, corroborating data from other investigators [16]. On the other hand, the prevalence of peritoneum and male genital morbidities was significantly higher in larger dogs, a finding not previously reported to the best of the author knowledge. This may be due to differences in the categorization of the organic systems; indeed, some authors bring together the genital and urinary tract under a broader designation of “urogenital system” [16].

In addition to represent an important morbidity, cardiovascular and urinary pathology also emerged as a frequent cause of death in elderly canines. However, despite the high prevalence of lesions in certain organic systems, there is not always a linear correspondence between them and the cause of death. Indeed, in spite of the high number of lesions identified in the reproductive system, their influence on the animal death is minor. Only 9.3% (7/75) of the reproductive system morbidities were determinant as cause of death. On the contrary, most of the lesions (7/9 = 77.8%) registered in the central nervous system were decisive to the animal death. Also 51.1% (24/47) of the mammary gland neoplasias and 39.3% (46/117) of the cardiovascular lesions represented a major cause of mortality.

In general, findings from this study parallel data obtained for human species, which display cancer and cardiovascular pathology as major causes of disease and death in older adults [1, 23, 24].

The main cause of death in geriatric canines population included in this study was neoplasia, which accounted for almost half of the deaths. This data is in accordance with results obtained by other authors, who described that death due to neoplastic disease increases with age [14–16, 25]. Moreover, larger dogs died of neoplastic disease more frequently than did smaller ones, a finding also referred by Fleming et al. [16]. Malignant neoplasms related to death mainly involved the mammary gland, followed by the hematopoietic and the digestive system. Bonnet and colleagues [13] also reported a high mortality rate attributed to mammary and hematopoietic neoplasia in a large population of dogs up to 10 years of age.

Old age and cardiac failure also represented important mortality causes, mirroring data published by Proschowsky et al. [11] and Adams et al. [14]. Neoplasia, along with cardiovascular failure and old age, were responsible for more than 80% of the deaths registered in our elderly canine population. On the other hand, traumatic lesions represented a minor cause of death in geriatric canines, which is in accordance with data published by other investigators [13, 15, 16] and parallels data obtained from humans [1].

Hyperplastic lesions (mostly registered in the prostate, liver, spleen and pancreas) and degenerative processes (especially involving the heart and the musculoskeletal system) were frequent morbidities found in this elderly dog population. Although not directly related to the animal death, they probably compromise several capabilities, contributing to the deterioration of its quality of life, leading the owners to request euthanasia for humanitarian reasons. Furthermore, animals included in the category “old age” may constitute a valuable model in the study of age-related multimorbidities, which afflicts a large proportion of human older adults, and besides of representing a challenge to clinical management, impose a serious overload on healthcare system [1, 26].

Results from this study are particularly relevant considering the One Health concept, a multidisciplinary and collaborative approach recognizing interconnections between humans, animals and their shared environment as crucial for achieving an optimal health outcome. The increase in human life expectancy observed in recent decades in developed countries, with inevitable higher demands on health services and threats to the pension systems sustainability, has justified the growing interest in understanding the mechanisms inherent to the aging process. Recently, some authors have emphasized the value of the dog as a suitable translational animal model for aging and gerontology [1, 8, 10]. Humans and canines share the same environment and are exposed to similar hazard contaminants and pollutants, carcinogenic stimuli, infectious agents and pro-inflammatory conditions [8, 10]. It is widely recognized that both develop a variety of similar spontaneous age-related morbidities and have access to similar medical and health care conditions [1, 8, 16]. Furthermore, canines’ longevity is about 6 to 7 times less than that of humans, which facilitates the collection of data in a short period of time [2]. In this sense, although there are morphofunctional differences between both species and in some diseases developed during the aging process, the dog can be regarded as a suitable translational model for human aging, meeting the concept of One Health.

Despite all the information obtained in this study, it is not exempt from some limitations. Although the age-related pathological processes categorized in this study were based on those of previous investigations, they are not entirely overlapping, which may explain some differences observed between our and other data already published. In fact, there is some variability in the definition of the pathological processes categories included in different studies. The canine population included in this study becomes from a specific geographic area (northern Portugal) and may not be entirely representative of the elderly dog population from other regions, making comparison with data from other studies difficult. Lack of information on the body condition score and on the animal age at the time of spaying/neutering did not allow the assessment of the relationship between these features and morbidity/mortality. On the other hand, some morbidities related to the central nervous and the musculoskeletal systems may have been underdiagnosed, since exhaustive assessment of these organic systems rely on the existence of clinical information suggestive of pathology (which is not always fully available). Much of the canines included in this study were euthanized upon the owner’s request, due to a deterioration in their quality of life and impairment of daily activities (such as incoordination and locomotor difficulties, inability to feed independently, incontinence and loss of vision). This was especially relevant in animals with old age or neoplasia, of which 92 and 85% were euthanized, respectively. This feature inevitably limits the value of the data obtained as an indicator of lifetime because, as an artificial shortening of lifespan, it does not measure how long the dogs could live, but still constitute very relevant information regarding the animal’s health span.

These results reinforce the potential of the domestic dog for further translational investigations on gerontology. Understanding the organic changes and morbidities inherent to aging represents an opportunity to the development of strategies for prevention, early diagnosis, and management of geriatric patients, ensuring longevity without compromising their quality of life. Insights into aging mechanisms allow the establishment of selective anti-aging interventions in the organic systems most affected by this biological process, providing an effective and more rationale use of resources. Understanding the complex mechanisms governing aging and longevity, as well as age-specific morbidity and mortality, is mandatory to retard the aging rate and extend a healthy lifespan, both in humans and animals [2, 5].

## Materials and methods

A total of 269 elderly dogs necropsied upon the owners’ request at the University of Porto, between 2012 and 2020, were included in this retrospective survey. Since geriatric checkups for small dogs are recommended by age 11, and for medium-sized ones at age 9–10 [27], dogs ≥10 years old were enrolled in this study. Moreover, knowing that canines’ longevity decreases with body size [25, 27–29], large dogs (over 20Kg) ≥ 8 years old were also included in the survey.

All the necropsies were performed by the same pathologist. Whenever necessary, samples were collected and fixed in 10% buffered formalin for histopathological examination. Necropsy reports were retrospectively reviewed and data regarding the identification of the animal, clinical history, macroscopic lesions observed at *post-mortem* inspection and corresponding histological examination were recorded. Morbidity was defined as the state of having a pathological condition, while mortality refers to death. Canines were divided in two groups according to their weight: ≤20 Kg and > 20Kg. The organ system affected was registered and partitioned into: cardiovascular, digestive, endocrine, genital, hematopoietic, liver, musculoskeletal, nervous, pancreas, peritoneum, respiratory, skin/subcutaneous and urinary.

The main pathologic process related to the animal death and type of death (natural vs euthanasia), was also recorded. Six major categories of pathologic processes related to mortality were defined: cardiovascular failure, inflammation, neoplasia, old age, trauma and others (see Glossary, at the end of the section, for the categories’ definition). Although several animals exhibited more than one pathological process (of the same or different categories) only the one that, due to its severity or bad prognosis, seems to be directly implicated in the animal death was considered when assigning this category. Thus, for example, a dog suffering from a pathological process of any type that died due to a traffic accident was included in the category “trauma”. . Although the motive of the animal death was known in cases of euthanasia, the underlying pathological condition that motivated the sacrifice request was considered as the main death-related cause.

Chi-square analysis was used to evaluate the significance of the relationship between categorical variables. Results were considered statistically significant for *p* values ≤0.05.

## Supplementary Information

**Additional file 1.**

## Data Availability

The datasets used and/or analysed during the current study available from the corresponding author on reasonable request.

## Glossary

Cardiovascular failure: includes heart and vascular lesions (eg. cardiomyopathies, cavitary hemorrhages)
Inflammation: includes inflammatory conditions regardless of the location (eg. pneumonia, nephritis, pyometra).
Neoplasia: includes only histologically confirmed malignant neoplasms
Old age: array of pathologic age-related changes impairing several organic systems, in which the severity of none of the lesions overlaps the others, so that the animal’s death cannot be attributed to a particular disorder nor to a specific system
Trauma: includes traumatic lesions regardless of the location
Others: includes lesions that could not be included in any of preceding categories
