# Supplementary material for: Morbidity and mortality in elderly dogs – a model for human aging
Source: BMC Vet Res. 2022 Dec 29;18:457. doi: 10.1186/s12917-022-03518-8 (PMC9798575; doi:10.1186/s12917-022-03518-8)
Supplement: Supplementary file 1 — Additional file 1. [file 12917_2022_3518_MOESM1_ESM.pdf]

Clinical necropsy request for teaching and research purposes

Sending institution<br><br>E-mail: ....<br>Tel..	Name: ....<br>Species: ....<br>Age: ....<br>Breed: ....<br>Gender: ....<br>Weight: ....

Clinical history/Anamnesis:

Known clinical history:____

____

Description of the clinical case/ lesions:

____

____

____

____

____

Veterinary clinician:

____

Animal tutor:

____

Note: All necropsy procedures are free of charge and do not incur any cost to the requester or tutor.
